# Two flagellar BAR domain proteins in *Trypanosoma brucei* with stage-specific regulation

**DOI:** 10.1038/srep35826

**Published:** 2016-10-25

**Authors:** Zdenka Cicova, Mario Dejung, Tomas Skalicky, Nicole Eisenhuth, Steffen Hanselmann, Brooke Morriswood, Luisa M. Figueiredo, Falk Butter, Christian J. Janzen

**Affiliations:** 1 Department of Cell & Developmental Biology, Biocenter, University of Würzburg, Würzburg, Germany; 2 Institute of Molecular Biology (IMB), Mainz, Germany; 3 Laboratory of Molecular Biology of Protists, Institute of Parasitology Biology Centre, Czech Academy of Science, and Faculty of Sciences, University of South Bohemia Ceske Budejovice, Czech Republic; 4 Instituto de Medicina Molecular, Faculdade de Medicina, Universidade de Lisboa, Lisboa 1649-028, Portugal

## Abstract

Trypanosomes are masters of adaptation to different host environments during their complex life cycle. Large-scale proteomic approaches provide information on changes at the cellular level, and in a systematic way. However, detailed work on single components is necessary to understand the adaptation mechanisms on a molecular level. Here, we have performed a detailed characterization of a bloodstream form (BSF) stage-specific putative flagellar host adaptation factor Tb927.11.2400, identified previously in a SILAC-based comparative proteome study. Tb927.11.2400 shares 38% amino acid identity with TbFlabarin (Tb927.11.2410), a procyclic form (PCF) stage-specific flagellar BAR domain protein. We named Tb927.11.2400 TbFlabarin-like (TbFlabarinL), and demonstrate that it originates from a gene duplication event, which occurred in the African trypanosomes. TbFlabarinL is not essential for the growth of the parasites under cell culture conditions and it is dispensable for developmental differentiation from BSF to the PCF *in vitro*. We generated TbFlabarinL-specific antibodies, and showed that it localizes in the flagellum. Co-immunoprecipitation experiments together with a biochemical cell fractionation suggest a dual association of TbFlabarinL with the flagellar membrane and the components of the paraflagellar rod.

The Kinetoplastida are a class of unicellular protists that share a common feature (the kinetoplast), comprising the mitochondrial DNA of the cell^1^. The class Kinetoplastida includes the order of exclusively parasitic Trypanosomatida, which are either monoxenous (restricted to one host individual), or dixenous (undergoing a complex life cycle between a host and a vector). Some species of this order are the causative agents of various infectious diseases distributed in many parts of the world, with a devastating impact on human health and the economies of impoverished countries. For example, *Leishmania donovani* causes visceral leishmaniasis (Kala-Azar) in South and Central America, South Europe, Africa and West Asia; *Trypanosoma cruzi* causes Chagas disease in South America; *Trypanosoma brucei gambiense* and *Trypanosoma brucei rhodesiense* are responsible for sleeping sickness in humans (human African trypanosomiasis, HAT), and *Trypanosoma brucei brucei* causes nagana in livestock in sub-Saharan Africa^2^. Both *Leishmania* and the two *Trypanosoma* species are transmitted to mammals by bloodsucking insects.

*T. brucei* has developed a complicated life cycle with different developmental stages in order to proliferate in mammalian hosts and to use the tsetse fly for dissemination. The *T. brucei* parasites first proliferate in the blood and adipose tissue of infected mammals as a long slender (LS) bloodstream form (BSF)^3^. The surface of the BSF parasites is covered by a densely packed variant surface glycoprotein (VSG) coat, which is central to the antigenic variation mechanism contributing to host immune system evasion^4^. Upon accumulation of a quorum sensing signal called stumpy induction factor (SIF)^5^, LS parasites differentiate into a cell cycle-arrested short stumpy (SS) form, which are pre-adapted for further differentiation in the tsetse fly. In the fly midgut, the SS form differentiates into a dividing procyclic form (PCF), the surface of which is covered with procyclin^6^. While PCFs migrate from the midgut they further differentiate and colonize the salivary glands as epimastigotes. Epimastigote trypanosomes are able to attach to the host microvilli of the epithelial cells lining the salivary glands lumen by the flagellar membrane flagellipodia^7^. While still attached to the salivary gland surface, trypanosomes acquire the VSG coat and mature into metacyclics. These cells are preadapted for transfer to and life in the mammalian host when the fly takes a next blood meal and completes the life cycle of the parasite^8^.

The mammalian host and the tsetse fly vector represent two completely different environments in terms of host immune response challenges, energy resources and temperature. The parasite has developed a sophisticated adaptation strategy and adjusts its morphology, motility, metabolism, gene expression and organelle activity to survive and proliferate in these different host environments. More recently, it was shown that even within the mammalian host, the parasites adapt to the different tissues, as parasites in the blood and adipose tissue are functionally different^3^. Understanding the host adaptation mechanisms of *T. brucei* during its life cycle has been a challenging task for the field. Several genome-wide transcriptome analyses have been performed to elucidate how trypanosomes adapt to different host environments. In addition to the comparison of transcript abundance in PCF and BSF^9^, the transcriptome of differentiating parasites has been analyzed^10^,^11^.

These studies provided many insights into the adaptation machinery of trypanosomes but there are certain limitations to transcriptome-based approaches. Due to the fact that the regulation of gene expression in trypanosomes occurs almost exclusively post-transcriptionally–at the level of mRNA stability, translational efficiency, and protein stability–the levels of mRNA do not always reflect the actual protein abundance in the cell^12^. For example, transcriptome-wide quantification of mRNA stability revealed that highly abundant transcripts in BSF have longer half lives compared to the same transcripts in PCF^13^. Furthermore, translational efficiency in PCF and BSF varies greatly between these two life cycle stages as shown by ribosome profiling^14^,^15^.

Hence, proteome-based studies are required to completely understand how the parasite changes during developmental differentiation. Recently, stable isotope labeling (SILAC) was used to quantitatively compare the proteomes of BSF and PCF, which elucidated many new components of the machinery for adaptation to the insect and mammalian hosts^16^,^17^,^18^. A previous study from our laboratories elucidated many new components of the host adaptation machinery^16^. A total of 4364 protein groups were analyzed and many new putative proteins of unknown function were detected. In all, 625 protein groups were enriched in the PCF and 253 protein groups were enriched in the BSF^16^. Furthermore, we also used label-free mass spectrometry techniques to quantify changes of the trypanosome proteome during stage differentiation from the mammalian-infective to the insect form^19^. This study revealed many previously unknown components of the differentiation machinery that are involved in essential biological processes such as signaling, post-translational protein modifications, trafficking and nuclear transport.

Large-scale proteomic studies are extremely useful to approach cellular changes in a systematic way. However, to fully understand the functions and consequences of differential gene expression, detailed work on a molecular level is necessary. We therefore decided to specifically characterize putative proteins of unknown function that are highly upregulated in the BSF to learn more about novel trypanosomal adaptation factors for the mammalian host. We therefore focused on a well-investigated structure that is highly adapted to different host environments: the trypanosome flagellum.

Trypanosomes have a single flagellum attached lengthwise along the cell body. The flagellum contains a canonical eukaryotic axoneme and a paracrystalline accessory structure termed the paraflagellar rod (PFR)^20^,^21^. The flagellum emerges from the flagellar pocket (FP), an invagination of the cellular membrane and the exclusive site of exo- and endocytosis^22^,^23^. The axoneme is nucleated by a barrel-like microtubule structure called the basal body, which abuts the FP membrane, while the PFR is initiated once the flagellum has exited the FP^24^,^25^. The flagellum is attached to the body of the cell by a flagellar attachment zone (FAZ). The FAZ is a tripartite, trans-membrane adhesion complex that links the axoneme and PFR to two structures within the cell that run underneath the attached flagellum^26^,^27^,^28^,^29^. These structures are the FAZ filament and a specialized microtubule quartet. The composition of the flagellum has been investigated in several proteomic studies in PCFs^30^.

A growing body of evidence indicates that the flagellum is a major communication hub with the host environment, providing sensing and response to extracellular signals. A combination of flagellum purification together with affinity purification of surface-exposed proteins identified flagellum matrix and surface proteins in the BSF and gave an insight into flagellum signaling^31^. Stage-specific expression of individual paralogs within gene families was demonstrated by comparison of PCF and BSF cell surface proteomes, showing that the parasite surface is remodeled to allow adaptation to the different host environments^32^. The flagellar membrane is in direct contact with the outer environment. In the epimastigote form of the parasite, found in the tsetse fly, it forms branched flagellar outgrowths that are attached to the salivary gland epithelium^33^. PCF stage-specific flagellar surface receptor adenylate cyclases have been shown to localize specifically to the flagellum tip. This supports subdomain organization of the flagellar membrane and a microdomain model for flagellar cyclic AMP (cAMP) signaling in *T. brucei*^34^. Several other signaling molecules, virulence factors or potential motility factors from the flagellum such as BSF expression site associated gene ESAG4, metacaspase 4, glycosylphosphatidylinositol-phospholipase C and calflagins were reviewed previously^34^,^35^. Arginine kinase 3 (AK3) is highly expressed in PCF compared to BSF and was shown to confer advantage to the parasites during infection in tsetse flies^36^. Interestingly, AK3 shares a similarity with the flagellar targeting sequence of calflagins from *T. brucei* and flagellar calcium binding protein (FCaBP) from *T. cruzi*^36^. Recently, a study of several trypanosome species suggested that flagellar motion and swimming behavior restricts the parasite to distinguishable anatomic niches within the mammalian hosts^37^. Taken together, it is critical to learn more about flagellar composition and function to fully understand how these parasites adapt to different environments during their complicated life cycle.

Here we describe a putative flagellar component, Tb927.11.2400, which was discovered in our comparative proteome study^16^. Database searches revealed that Tb927.11.2400 shares 38% amino acid identity with PCF-specific *Tb*Flabarin (Tb927.11. 2410). Flabarin was initially described in *Leishmania donovani* as a flagellar BAR domain protein^38^*. Ld*Flabarin is targeted to the flagellum by a potential N-terminal acylation site and has a central BAR domain for membrane association and a C-terminal domain needed for the flagellar specificity. It forms a helicoidal structure from the base to the tip of the flagellum^38^. *In vitro* experiments suggest a morphogenetic and structural function since recombinant *Ld*Flabarin associates with liposomes and triggers tubule formation^38^. We named Tb927.11.2400 *TbFlabarin-Like* (*Tb*FlabarinL) following the convention adopted for Flabarins^38^. The two homologues in trypanosomes are likely a result of a gene duplication event. *Tb*FlabarinL is downregulated 24 hours post induction of differentiation and undetectable in PCF. In contrast, *Tb*Flabarin is upregulated very early during the differentiation process (2 h after induction) and is fully expressed 24 hours post induction. Protein expression levels of *Tb*FlabarinL are 20-fold higher in BSF compared to PCF, which suggest that this protein might be a potential mammalian host-specific adaptation factor. We showed that *Tb*FlabarinL is not essential for the growth of the parasite under cell culture conditions and that it is dispensable for developmental differentiation of trypanosomes. We generated a *Tb*FlabarinL specific antibody and show that the endogenous protein localizes to the flagellum. We found that *Tb*FlabarinL associates with both the flagellar membrane as well as with the PFR.

## Materials and Methods

### Computational analyses

A BLAST search with *Tb*FlabarinL (Tb927.11.2400) was performed against the TriTryp database^39^. Alignments were obtained from constraint-based multiple alignment tool (COBALT)^40^ at the NCBI database and edited in Espript 3.0^41^. Protein structures of *Tb*FlabarinL and *Tb*Flabarin were predicted by the Phyre2 server^42^ and edited in UCSF Chimera 1.10.2^43^. Datasets for phylogenetic analyses were received from publicly available sources (Supplementary Table 1) for both Flabarin and FlabarinL genes, using BLASTP at an E-value cut-off of 10–20. All amino acid sequences were searched for conserved domains by Pfam. The datasets were aligned by MUSCLE and relevant positions were selected using Gblocks. Phylogenetic model selection with Modelgenerator favored LG + GAMMA model and ML trees were constructed using RAxML 8.1.17 with 1 000 bootstrap replicates. Bayesian Monte Carlo Markov (MCM) chain analysis was performed with GTR + GAMMA + CAT model using Phylobayes 3.3f running 8 independent chains for 10,000 cycles. Convergence of chains was estimated by comparison of bipartition frequencies in individual chains, discarding first 2,000 cycles.

### Trypanosome cell lines and cultivation

Monomorphic BSF trypanosome “Single Marker” (SM) and 2T1 are both derivatives of Lister strain 427, antigenic type MITat 1.2, clone 221a (Doyle et al., 1980) and express T7 polymerase and Tetracycline repressor^44^. Monomorphic BSF trypanosomes were maintained in HMI-9 medium with 10% fetal calf serum (Sigma) and 5% CO_2_^45^. PCF trypanosomes were cultured in modified SDM-79 medium with 10% fetal calf serum (Sigma) and 5% CO_2_^46^. BSF and PCF cell densities were determined using a Coulter Counter Z2 (Beckman Coulter) particle counter, and cultures were diluted to maintain the cells in mid log growth phase. Transfection of monomorphic trypanosomes was performed as described previously^47^. Pleomorphic BSF trypanosomes AnTat1.1 were cultured in HMI-9 medium containing 1.1% methylcellulose. Cell density was determined using a Neubauer counting chamber (Brand) and cultures were diluted to maintain the cells in mid log growth phase. Cells were transfected as described previously^48^,^49^.

### Differentiation of trypanosomes

Pleomorphic AnTat1.1 BSF were grown to a density of 2.5 × 10^6 ^cells/ml, diluted 1:5 in TDB (5 mM KCl, 80 mM NaCl, 1 mM MgSO_4_, 20 mM Na_2_HPO_4_, 2 mM NaH_2_PO_4_, 20 mM glucose, pH 7.4), filtered, sedimented by centrifugation (1500 × g, 10 min, 37 °C) and resuspended to a cell density of 2 × 10^6 ^cells/ml in DTM medium^50^. A mixture of cis-aconitate and isocitrate (3 mM) was added to induce differentiation and cells were incubated at 27 °C with 5% CO_2_ as described previously^50^.

### Generation of transgenic trypanosome cell lines

To generate the *Tb*FlabarinL^*RNAi*^ cell line, a DNA fragment (positions 30–544) was amplified from genomic DNA using a forward primer containing SmaI and XhoI restriction sites and a reverse primer containing BamHI and XbaI sites. Sense and antisense fragments of the RNAi hairpin were ligated into the pRPAiSL vector^51^. Prior to transfection of 2T1 cells, the plasmids were linearized using AscI. Clones with a correctly integrated construct were selected as described previously^51^. RNAi was induced by addition of 1 μg/ml tetracycline to the cell culture and refreshed daily.

A PCR-based gene deletion approach was used to sequentially replace both alleles of *Tb*FlabarinL with puromycin N-acetyl-transferase (PUR) and hygromycin phosphotransferase (HYG) open reading frames (ORFs) in MiTat1.2 SM. The ends of the *Tb*FlabarinL 5′ UTR (59 nt) and 3′ UTR (60 nt) were flanked by sequences to amplify the *HYG* and *PUR* ORFs from the pHD309 HYG/PUR plasmid (gift from G.A.M. Cross). Cells were electroporated with the purified PCR products. The same constructs were used to delete both *TbFlabarinL* alleles in AnTat1.1 and AnTat1.1E-SmOx cells. Correct integration of the constructs was verified by PCR using primers binding in the 5′ and 3′ UTR, 5′ and 3′ ORF and within the puromycin and hygromycin resistance ORFs (Supplementary Figs S3 and S4). PCR was performed according to the manufacturer’s instructions of the Phusion Human Specimen Direct PCR Kit (Thermo Fisher Scientific).

*Tb*FlabarinL was epitope-tagged at the N- or C-terminus in the endogenous locus as described in ref. 52. The plasmids for tagging were kindly provided by S. Kramer, University of Würzburg. Briefly, the *Tb*FlabarinL ORF fragment without stop codon was amplified from genomic DNA and cloned into p3077_*PAC*_4xTY plasmid. The vector was linearized within the ORF fragment by HpaI prior to transfection of the cells. To tag *Tb*FlabarinL at the C-terminus, a part of the *Tb*FlabarinL ORF (positions 2-653) was amplified from genomic DNA and cloned into p3074_4xTY_*BLE* plasmid and linearized by HpaI before transfection of the cells. The same strategy was used to tag *Tb*Flabarin (Tb927.11.2410) at the C-terminus. A fragment of the *Tb*Flabarin ORF (positions 1-666) was cloned into p3074_4xTY_*BLE* plasmid and linearized with BclI enzyme prior transfection. To tag PAR1 (Tb927.11.13500) at the N-terminus, a fragment (positions 4-823) of the PAR1 ORF was cloned into the p3077_*PAC*_4xTY plasmid and EcoRI was used for linearization.

To express *Tb*FlabarinL in PCF ectopically, its ORF was amplified from genomic DNA and cloned into pLEW100v5b2x_Phleo (gift from G.A.M. Cross) using XhoI and HindIII restriction sites. For ectopic expression of *Tb*Flabarin in BSF, the *Tb*Flabarin ORF was amplified from genomic DNA using a C-terminal primer containing a glycine-alanine-glycine (Gly-Ala-Gly) linker sequence followed by a Ty1-tag sequence and cloned into pLEW100v5b2x_Phleo using XhoI and HindIII restriction sites. Constructs were linearized with NotI for integration into the ribosomal spacer locus. Expression was induced by addition of 1 μg/ml tetracycline to the cell culture.

### Polyclonal Antibody Generation

The *Tb*FlabarinL ORF was amplified from genomic DNA of *T. brucei* using a primer containing a 10x His-tag sequence and a Gly-Ala-Gly linker sequence and cloned into the pETDUET-1 vector (Novagen) using NcoI and XhoI restriction sites. *Tb*FlabarinL was expressed in Rosetta Blue *Escherichia coli* according to the manufacturer’s instructions and purified using an Äkta FPLC system with HisTrap FF crude 1 ml columns. Fractions containing recombinant *Tb*FlabarinL were pooled, dialyzed in phosphate buffered saline and concentrated using a centrifugal filter unit with a 10 kDa cut off (Amicon). 1 mg of purified protein was sent to Pineda (Berlin) for antibody production. *Tb*FlabarinL-specific antibody was affinity purified from the rabbit immune sera using the SulfoLink kit (Thermo Fisher Scientific) and recombinant *Tb*FlabarinL according to the manufacturer’s instructions. Antibody specificity was tested in immunoblots using recombinant *Tb*FlabarinL and whole-cell lysates of wild-type, ∆*Tb*FlabarinL and *Tb*FlabarinL^*RNAi*^ cell lines.

All primer sequences used in this study are available upon request.

### Western Blot (WB)

Whole-cell lysates were separated by SDS-PAGE on 10% polyacrylamide gels and transferred onto a Immobilon® PVDF membrane (MERCK MILLIPORE). The membrane was blocked 1 h at RT in PBS-5% milk and subsequently incubated 1 h at RT with polyclonal anti-*Tb*FlabarinL rabbit antibody diluted 1:500 in PBS-1% milk. Monoclonal anti-PFR mouse antibody L13D6, monoclonal anti-tubulin TAT1 mouse antibody and BB2 anti-Ty1 mouse antibody were gifts from K. Gull (University of Oxford) and described elsewhere^53^,^54^,^55^. Anti-TbMORN1 rabbit antibody was described previously^56^. IRDye680- and IRDye800-coupled anti-mouse or anti-rabbit secondary antibodies were purchased from LI-COR Bioscience to detect the respective proteins with an Odyssey infrared imaging system (LICOR Bioscience).

### Immunofluorescence (IF)

1 × 10^7^ cells were harvested by centrifugation (1500 × g, 10 min, RT) and resuspended in 1 ml TDB. Cells were fixed in 2% PFA (10 min, RT). After 3 wash steps in PBS, cells were settled onto Poly-L-lysine-coated slides (20 min, RT) and permeabilized using 0.2% IGEPAL in PBS (5 min, RT). Non-specific epitopes were blocked with 1% BSA in PBS (1 hr at 37 °C). Cells were incubated with the primary *Tb*FlabarinL antibody diluted 1:200 in PBS-0.1% BSA and/or monoclonal BB2 anti-Ty1 mouse antibody diluted 1:500 in PBS-0.1% BSA (1 hr, RT). After washing with PBS, the secondary antibody was applied (Alexa 594 anti-mouse, Alexa 488 anti-rabbit (Life Technologies)) in PBS-0.1% BSA (30 min, RT). DNA was stained with 1 μg/ml 4,6-diamidino-2-phenylindole (DAPI) and cells were embedded in ProLong Gold Antifade (Molecular Probes) and imaged on Leica DMI 6000B microscope. Images were processed using Huygens Essential XII deconvolution software (Scientific Volume Imaging).

### Co-immunoprecipitation

Polyclonal *Tb*FlabarinL antibody was immobilized on protein G Sepharose Fast Flow beads (GE Healthcare). Non-specific binding was blocked (1 hr, 4 °C) with 0.5% BSA in 20 mM sodium phosphate buffer pH 7.0. Four biological replicates per cell line (2 × 10^8^ cells each) were harvested by centrifugation (1500 × g, 10 min, 4 °C) and washed in TDB buffer. Cells were lysed in 400 μl IP buffer (150 mM NaCl, 20 mM Tris-HCl pH 8, 10 mM MgCl_2,_ 0.25% IGEPAL 1 mM DTT, Protease Inhibitors Cocktail without EDTA (Roche)) by sonication using a Bioruptor (Diagenode) with a 3 cycles 30 s high power pulse with 30 s pause setting. Cell lysates were incubated with the blocked beads (orbital mixing, overnight, 4 °C). Beads were washed in IP buffer. To elute the proteins, beads were resuspended in 65 μl NuPAGE LDS Sample Buffer (Novex, Life Technologies) supplemented with 100 mM DTT and incubated 10 min at 70 °C. The eluates were analyzed by mass spectrometry.

### Mass Spectrometry

Protein samples were separated on a 4–12% NuPAGE Gel (Life Technologies) and stained with Coomassie colloidal blue (Life Technologies). The lanes were sliced and prepared by in-gel digestion with trypsin^57^. The peptides were stored on StageTips. Digested peptides were separated on a C18 reverse phase column (packed in-house, 20 cm, 75 μm inner diameter; packed with reprosilPur-1.8 [Dr. Maisch]) with a 105-minute gradient from 5 to 95 percent ACN on an Easy-nLC 1000. The solution was directly sprayed at 2.4 kV. The Q Exactive Plus was operated in a data-dependent Top10 acquisition mode with one full scan (70,000 resolution, max injection 20 ms, 300–1650 m/z) and up to 10 HCD fragment scans (17,500 resolution, max injection 120 ms). The raw spectra were analysed with MaxQuant ver1.5.1.0^58^ using standard settings (except activated LFQ quantitation and match between runs) and the *Trypanosoma brucei* TriTrypDB-8.0_TbruceiTREU927 database. The protein Groups file was filtered to exclude contaminants, reverse entries and proteins only identified by site prior to statistical analysis. To obtain enrichment the LFQ values were log2 transformed and the mean between bait and control was calculated. To assess the statistical significance of the enrichment a Welch t-test for LFQ values between bait and control set was performed. Both values were visualized in a volcano plot using the ggplot2 package in R.

### Mouse experiments

Animal experiments were performed according to EU regulations and approved by the Animal Ethics Committee of Instituto de Medicina Molecular (AEC_2011_006_LF_TBrucei_IMM). Male C57BL/6 wild-type mice (Charles River) were housed in the pathogen-free mouse facility of the Instituto de Medicina Molecular (IMM) with a 12:12-h light:dark cycle. 6 weeks-old mice were used for intraperitoneal infection with 2 × 10^3^ parasites each. 5 mice were infected with *T. brucei* AnTat1.1E-SmOx parasites and 6 mice with the ∆*Tb*FlabarinL cell line. Parasitemia was measured after infection by collecting blood from the tail vein. The vein was punctured with a gauge needle and 1 μl of blood was collected and diluted in 149 μl HMI-11. Parasites numbers were manually quantified using a Neubauer counting chamber. The minimum of detectable parasites in blood is 1.5 × 10^5 ^cells/ml.

### Isolation of cytoskeletons and flagella

Cytoskeleton- and flagella-enriched fractions were prepared following a published protocol^59^,^60^ with minor modifications. Briefly, 1×10^8^ cells were harvested by centrifugation (1500 × g, 10 min, 4 °C) and washed in 1 ml PEME buffer (2 mM EGTA, 1 mM MgSO4, 0.1 mM EDTA, 0.1 M piperazine-N,N′-bis (2-ethanesulfonic acid)-NaOH (PIPES-NaOH), pH 6.9). Cytoskeletons (P1) were obtained by extracting the cells in PEME buffer with 1% IGEPAL (15 min, RT, orbital mixing) and separated from the detergent soluble supernatant (S1) by centrifugation (3400 × g, 5 min, 4 °C). Flagella were isolated from the extracted cytoskeletons (P1) by incubation in PEME buffer with 1% IGEPAL and 1M KCl (30 min, RT, orbital mixing), which depolymerized the corset microtubules, followed by centrifugation (6000 × g, 15 min, 4 °C) to separate S2 and P2 fractions. All solutions were supplemented with Complete protease inhibitors cocktail without EDTA (Roche).

## Results

### *Tb*FlabarinL originated from a gene duplication event

**Figure 1:**
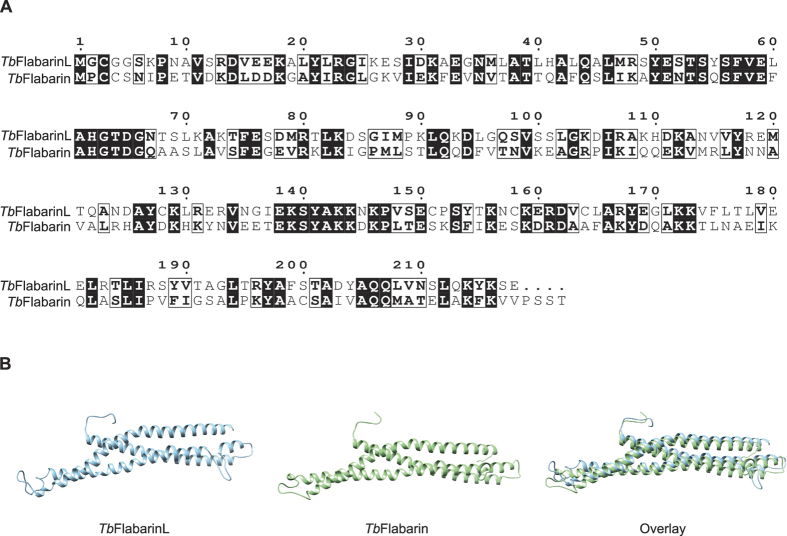
**(A)** Alignment of *Tb*FlabarinL (Tb927.11.2400) with *Tb*Flabarin (Tb927.11.2410). A BLAST search with the Tb927.11.2400 protein sequence identified a *Trypanosoma brucei* flabarin homologue (Tb927.11.2410). The amino acid sequences of *Tb*FlabarinL and *Tb*Flabarin have 38% identity (indicated by black boxes) and 60% similarity (indicated by white boxes). **(B)** Sequence-based structure modeling of *Tb*FlabarinL and *Tb*Flabarin. An overlay of the two structures is shown.

*Tb*FlabarinL (Tb927.11. 2400) was identified in a proteomic study^16^ as a BSF-specific, putative protein of unknown function. A BLAST search in the TriTryp database^39^ revealed a 38% amino acid (AA) identity and 60% similarity^61^,^62^ to the product of the neighboring Tb927.11.2410 gene (Fig. 1A). Tb927.11.2410 is *Tb*Flabarin, a flagellar BAR domain protein in *T. brucei*. Flabarins were first described in *Leishmania spp*^38^. *Tb*Flabarin consists almost entirely of a BAR/IMD-like domain, which extends over the region of 8–212 AA out of a total 222 AA length of the protein. Despite the high similarity to *Tb*Flabarin (60% AA similarity), a BAR/IMD like domain was not detected by the prediction software in *Tb*FlabarinL. However, predicted structural models^42^ of both *Tb*Flabarin and *Tb*FlabarinL feature a triple helix coiled coil architecture (Fig. 1B). Such a structure is characteristic of BAR proteins and enables formation of banana-shaped homo- or heterodimers known for their membrane curvature generation (reviewed in ref. 63).

**Figure 2:**
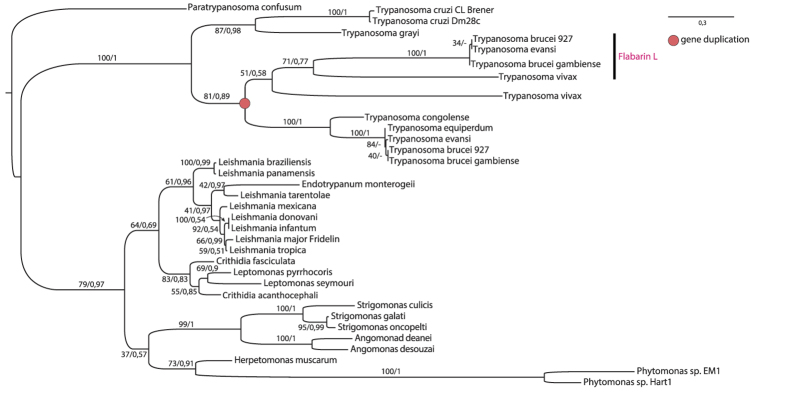
Phylogenetic analysis of Flabarins across Trypanosomatida. A maximum likelihood phylogenetic tree based on alignments of Flabarin and FlabarinL proteins. The scale bar indicates the inferred number of amino acid substitutions per site. The red dot indicates a gene duplication event that gave rise to the FlabarinL gene, which is present only in a subset of African trypanosomes.

*Tb*Flabarin and *Tb*FlabarinL are both found on chromosome 11 separated by an ~12 kb stretch of non-coding sequence. The *Tb*Flabarin coding sequence (CDS) is on the complementary DNA strand. Orthologs of *Tb*FlabarinL are present within the orthology group OG5_185161 from Ortho MCL DB^64^,^65^ in *T. brucei gambiense*, *T. vivax* and *T. evansi*. In contrast to Flabarin OG5_148786, FlabarinL is neither present in the genomes of sequenced monoxenous (one-host) trypanosomatids, nor in the genus *Leishmania* and American trypanosomes. Phylogenetic analysis revealed a gene duplication event in African trypanosomes that gave rise to the FlabarinL gene (Fig. 2). Interestingly, FlabarinL is missing in *T. congolense* and *T. equiperdum*, which might be due to incompleteness of these genomes. On the other hand, it could reflect specific host adaptation requirements.

**Figure 3:**
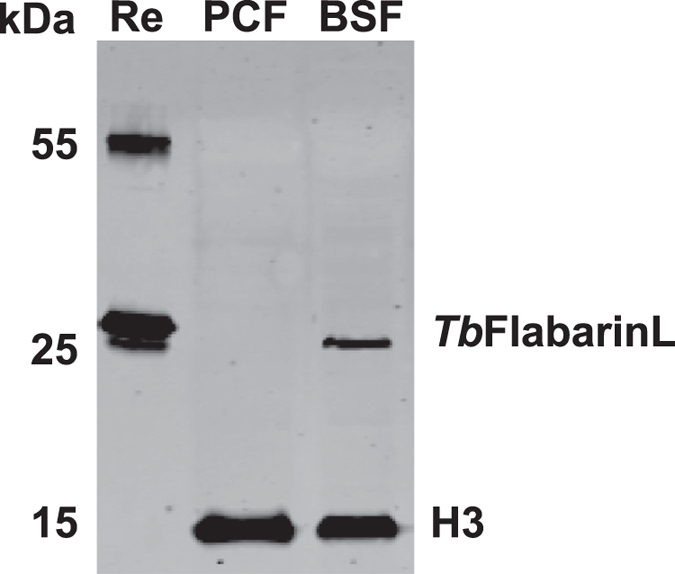
*Tb*FlabarinL is a BSF stage-specific protein. Immunoblot analysis of whole cell lysates from PCF and BSF cells using affinity purified anti-*Tb*FlabarinL antibodies. Expression of *Tb*FlabarinL was detected in BSF cells only. Immunoblotting with anti-Histone H3 served as a loading control. A representative blot of multiple (N > 3) independent experiments is shown. Re: recombinant His-tagged TbFlabarinL.

To confirm the expression profiles of the mass spectrometry study, we generated a *Tb*FlabarinL-specific antibody. Recombinant full-length *Tb*FlabarinL was purified from *E. coli* (Supplementary Fig. S1) and used for immunization of a rabbit. *Tb*FlabarinL-specific antibody was affinity purified from the immune sera and used in Western blot (WB) analysis (Fig. 3). A band of the expected molecular weight could be detected in BSF whole cell lysates but not in lysates from PCF, supporting the conclusion from mass-spectrometry analysis that *Tb*FlabarinL is a BSF stage-specific protein.

### *Tb*FlabarinL localizes to the flagellum in BSF trypanosomes

**Figure 4:**
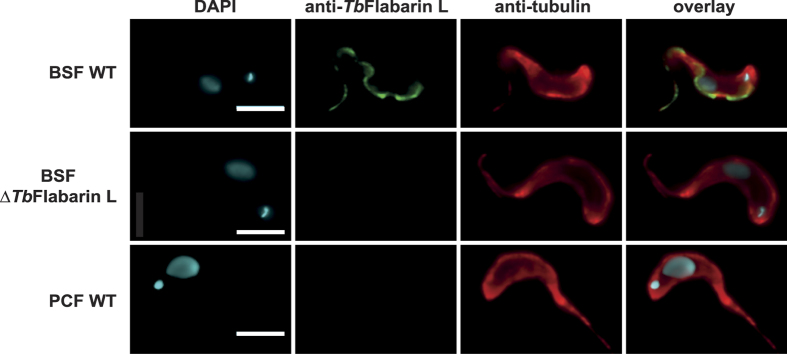
*Tb*FlabarinL is localized to the flagellum. Immunofluorescence analysis using the anti-*Tb*FlabarinL antibody showed that *Tb*FlabarinL localized to the flagellum of wild-type (WT) *T. brucei* BSF cells. No labeling pattern was observed in the BSF knockout cell line (BSF Δ*Tb*Flabarin L) or PCF WT cells Scale bar 3 μm.

*Tb*FlabarinL-specific antibody was used in immunostaining to determine the subcellular localization of *Tb*FlabarinL in BSF trypanosomes. A ∆*Tb*FlabarinL cell line (described in later sections) and PCF cells were used as controls. Cells were stained with *Tb*FlabarinL-specific antibody and TAT1 anti-tubulin antibody (Fig. 4). *Tb*FlabarinL localized to the flagellum in BSFs, with a labeling pattern that initiated close to the kinetoplast (small spot in DAPI channel) and extended lengthwise along the cell body. No signal was detected from the ∆*Tb*FlabarinL cells, confirming the specificity of the labeling pattern. No signal was detected in PCF cells, further confirming the stage-specific expression of the *Tb*FlabarinL protein.

**Figure 5:**
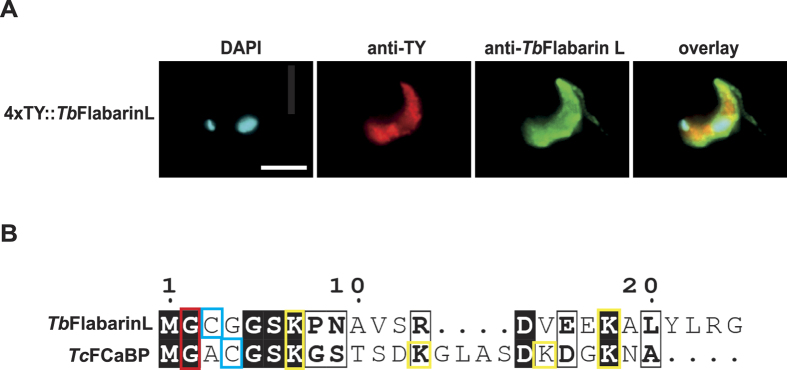
*In situ* N-terminal tagging results in mislocalization of *Tb*FlabarinL to the cytoplasm. (**A**) The N-terminally Ty1-tagged *Tb*FlabarinL (red) shows localization in the cytosol whereas the *Tb*FlabarinL WT protein (green) also localizes to the flagellum. The *Tb*FlabarinL-specific antibody recognizes both the WT and the tagged allele of *Tb*FlabarinL. Scale bar 3 μm. **(B)** The N-terminus of *Tb*FlabarinL is similar to N-terminal sequences of Flagellar Calcium Binding Protein (FCaBP) of *T. cruzi*. Elements potentially necessary for the association with the flagellar membrane are highlighted. Palmitoylated and myristoylated amino acids are marked in red and blue, respectively. Conserved lysines are marked in yellow. Identical and conserved residues are boxed in black and white respectively.

We observed that N-terminal tagging of *Tb*FlabarinL resulted in a mislocalization of *Tb*FlabarinL. Cells in which the ORF of only one allele was fused to a sequence that encodes for a 4xTy1 epitope tag by homologous recombination were stained with BB2 anti-Ty1 antibody and *Tb*FlabarinL specific antibodies. The tagged version of *Tb*FlabarinL remained in the cytoplasm (Fig. 5A), whereas the wild-type version of could be detected in the flagellum. Interestingly, the first 24 N-terminal AA of *Tb*FlabarinL share 75% identity with the N-terminal sequence of flagellar calcium binding protein (FCaBP) of *T. cruzi* (Fig. 5B). The first 24 AA are responsible for flagellar localization and for the association of FCaBP with the inner leaflet of the flagellar membrane^66^. These effects are mediated by the presence of a myristoylated glycine in position 2, palmitoylated cysteine in position 4, and two conserved lysines, *Tb*FlabarinL contains glycine in position 2 and cysteine in position 3 and two positively charged lysines. The similarity of the N-termini of *Tb*FlabarinL and FCaBP and mislocalization of *Tb*FlabarinL after *in-situ* N-terminal tagging implies *Tb*FlabarinL flagellar membrane localization is probably mediated by similar targeting mechanisms.

**Figure 6:**
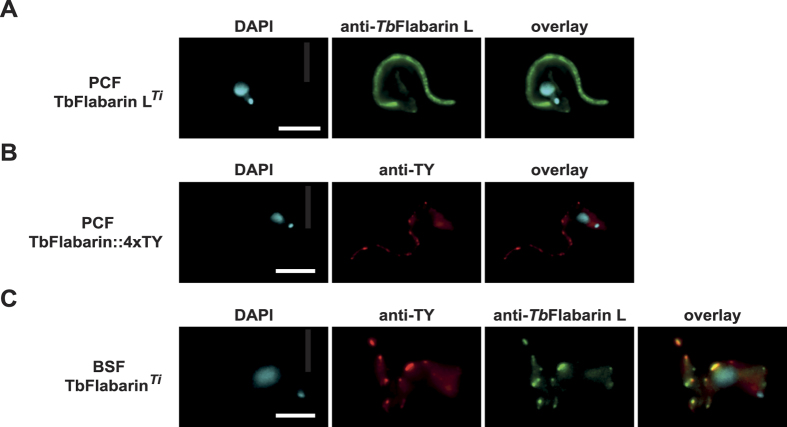
*Tb*Flabarin and *Tb*FlabarinL localization in the BSF and PCF *T. brucei.* Immunofluorescence staining using anti-Ty1 (red) and anti-*Tb*FlabarinL (green) antibodies showed that ectopically expressed *Tb*Flabarin in BSF had a similar patchy localization pattern as *Tb*FlabarinL. The localization of the two proteins overlaps in some regions. **(B)** Ectopically expressed *Tb*FlabarinL localized to the flagellum in PCF. **(C)** *In situ* C-terminally tagged *Tb*Flabarin in PCF showed a similar localization pattern in the flagellum to the one of *Tb*FlabarinL in BSF. Scale bar 3 μm.

To analyze the similarity of *Tb*FlabarinL and *Tb*Flabarin, and to test if a common protein targeting mechanism apply for the procyclic protein, C-terminally Ty1 epitope-tagged *Tb*Flabarin was ectopically expressed in BSF. *Tb*Flabarin localized clearly to the flagellum with a discontinuous pattern similar to that of *Tb*FlabarinL. Staining with anti-Ty1 and anti-*Tb*FlabarinL antibodies showed an overlap of the signal in distinct regions of the flagellum (Fig. 6A). Conversely, ectopically overexpressed *Tb*FlabarinL in PCF localized to the flagellum in a more continuous pattern along the whole length of the flagellum (Fig. 6B) presumably due to high expression levels (200-fold overexpression compared to endogenous protein levels in PCF). *In situ* C-terminally Ty1-tagged *Tb*Flabarin in PCF localized to the flagellum and displayed a patchy pattern similar to that of *Tb*FlabarinL in BSF (Fig. 6C). These observations are consistent with the hypothesis that *Tb*FlabarinL and *Tb*Flabarin have a similar stage-specific function in the flagellum of *T. brucei.*

### *Tb*FlabarinL is associated with the paraflagellar rod and the flagellar membrane

**Figure 7:**
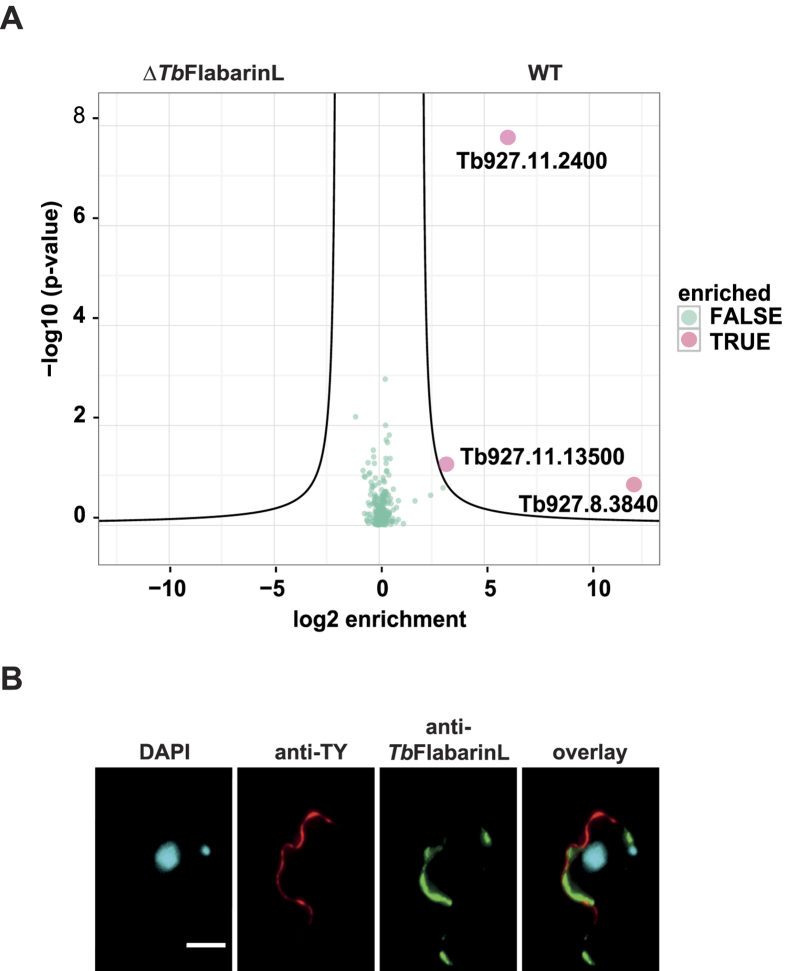
Immunoprecipitation using *Tb*FlabarinL specific antibody. (**A**) Volcano plot of co-enriched proteins after anti-*Tb*FlabarinL IP obtained by label-free quantitative mass spectrometry of four parallel biological replicates. Besides *Tb*FlabarinL (Tb927.11.2400), two other proteins were enriched: Tb927.11.3840 and Tb927.11.13500. Tb927.11.3840 is an uncharacterized hypothetical protein. Tb927.11.13500 is PAR1, a paraflagellar rod component. (**B**) *Tb*FlabarinL partially overlaps with the PFR component Par1 in the flagellum. Immunofluorescence analysis using the *Tb*FlabarinL specific antibody showed that *Tb*FlabarinL localized to the flagellum of *T. brucei*. A co-staining of the PFR component PAR1 (Ty1-tagged, red) and anti-*Tb*FlabarinL (green) antibody showed *Tb*FlabarinL and PAR1 overlap in certain regions of the flagellum. Scale bar 3 μm.

To learn more about the function of *Tb*FlabarinL, we employed a co-immunoprecipitation (IP) approach to find interacting partners of *Tb*FlabarinL. Four biological replicates of WT and ∆TbFlabarinL as a control were used in IP and analyzed by mass spectrometry. Two putative interacting partners Tb927.11.3840 and Tb927.11.13500 (Fig. 7A) were found. The first candidate interaction partner is an unknown putative protein with no conserved domains. The second candidate interaction partner is PAR1, a previously-described paraflagellar rod component^67^,^68^,^69^. To verify the result of the IP, an endogenous replacement cell line was generated by homologous recombination, with the PAR1 ORF fused to a sequence that encodes for a 4xTy1 epitope tag. These cells were stained with the anti-Ty1 antibody for immunolocalization studies. As expected, 4xTy1-PAR1 localized to the flagellum (Fig. 7B). Detection with anti-Ty1 and *Tb*FlabarinL specific antibodies showed a partial overlap of *Tb*FlabarinL and Ty1-PAR1 in distinct parts of the flagellum, which supported the result of the IP. Interestingly, *Tb*FlabarinL localization revealed a different pattern compared to PAR1. *Tb*FlabarinL showed an interrupted and punctate pattern throughout the flagellum (Fig. 7B).

**Figure 8:**
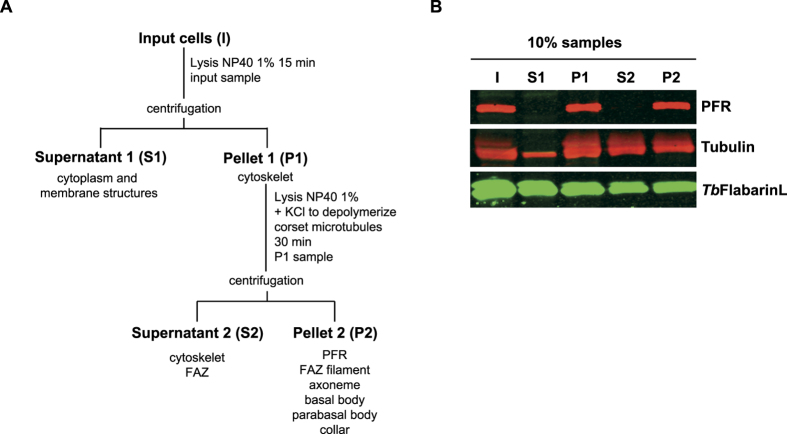
Biochemical cell fractionation. **(A)** Fractionation scheme: The input sample (I) was taken after non-ionic detergent extraction of BSF cells. The cells were then separated by centrifugation into a detergent-soluble supernatant (S1) and detergent-insoluble cytoskeletal pellet (P1). The P1 fraction was solubilized in high salt to depolymerize corset microtubules and further separated by centrifugation into supernatant (S2) and pellet (P2) fractions. **(B)** Fractionation of *Tb*FlabarinL. Equal fractions (10%) were loaded on a gel and analyzed by immunoblotting. Anti-PFR, anti-tubulin and *Tb*FlabarinL specific antibodies were used. *Tb*FlabarinL was found in all fractions.

The results of the IP experiment as well as the partial overlap of PAR1 and *Tb*FlabarinL immunostaining imply a possible association of *Tb*FlabarinL with the PFR structure of the flagellum. In contrast, the N-terminal sequence of *Tb*FlabarinL seems to attach *Tb*FlabarinL to the flagellar membrane. To probe the membrane association of *Tb*FlabarinL, a biochemical cell fractionation was performed. In the first step, the detergent soluble fraction S1 (cytoplasm/membranes) was separated from P1 (cytoskeleton). In the second step, the cytoskeletal P1 fraction was treated with 1 M KCl to depolymerize the microtubule corset and the FAZ filament and thus separate S2 (corset microtubules together with FAZ) from P2 (PFR, axoneme, basal body and flagellar pocket collar). WB analysis of equal fractions (10% of input material) revealed that *Tb*FlabarinL is found not only within the soluble cytoplasmic and membrane fractions but also within the cytoskeletal fractions both before and after separation of the flagella from the cytoskeleton (Fig. 8). This result supports the observations described in previous experiments, which suggested a dual interaction with the flagellar membrane as well as with structural components of the flagellum such as the PFR.

### *Tb*FlabarinL is not essential for BSF *in vitro* and *in vivo*

**Figure 9:**
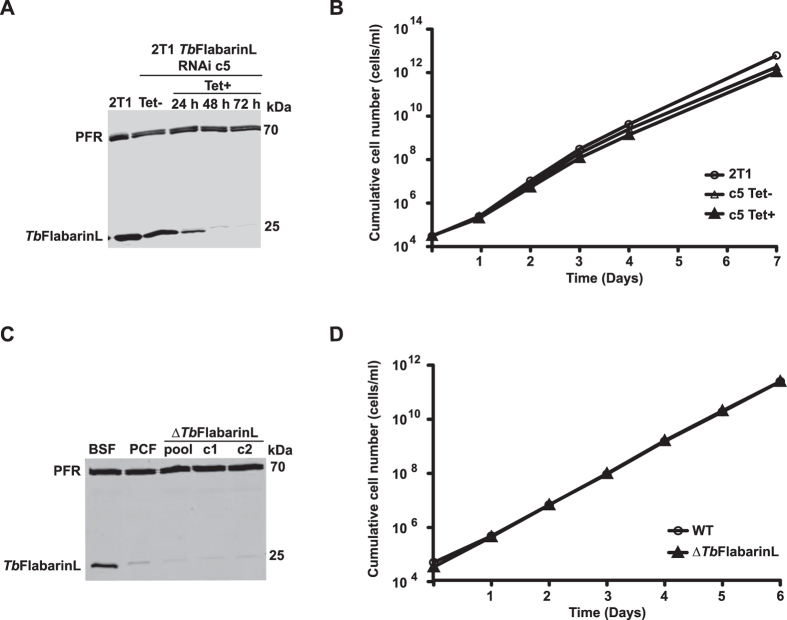
Depletion of *Tb*FlabarinL in BSF *T. brucei*. **(A)** *Tb*FlabarinL protein was almost undetectable 48 h after induction of RNAi, as determined by anti-*Tb*FlabarinL immunoblot. A weak additional band with the size of 25 kDa is detectable after RNAi induction. PFR was used as a loading control. **(B)** Depletion of *Tb*FlabarinL by RNAi had no effect on the growth of BSF *T. brucei* under cell culture conditions. A cumulative growth curve of one representative clone is shown. Two independent clones showed a similar phenotype (see Supplementary Fig. S2). **(C)** An immunoblot analysis using *Tb*FlabarinL specific antibody confirmed deletion of *Tb*FlabarinL in two independent clones (c1 and c2) and a non-clonal population (pool). A weak additional band with the size of 25 kDa is detectable after deletion of *Tb*FlabarinL. Immunoblotting with anti-PFR antibodies was used as a loading control. **(D)** The ∆*Tb*FlabarinL cells showed no growth defect compared to the wild-type controls (N = 3).

In order to examine the effect of *Tb*FlabarinL depletion on the viability of *T. brucei*, an inducible RNAi knockdown cell line was generated. *Tb*FlabarinL expression was significantly reduced 24 h after induction and almost below detection level after 48 h (Fig. 9A). The growth of the parasites upon induction of RNAi was monitored. There was no growth phenotype observed upon depletion of *Tb*FlabarinL in three independent clones (Fig. 9B and Supplementary Fig. S2), indicating that *Tb*FlabarinL is not an essential gene for the survival of the parasite under cell culture conditions. To further pursue this indication, a ∆*Tb*FlabarinL knockout cell line was generated in BSF trypanosomes by homologous recombination. The deletion of both *TbFlabarinL* alleles was verified by integration PCR (Supplementary Fig. S3) and by WB analysis (Fig. 9C). No difference was observed in growth between the parental and the ∆*Tb*FlabarinL cell lines (Fig. 9D). To test the role of *Tb*FlabarinL *in vivo*, we infected mice with WT and ∆*Tb*FlabarinL pleomorphic cell lines. We detected no significant differences between the parasitemia profiles and survival curves, suggesting that deletion of *Tb*FlabarinL has no effect on mammalian infections (Fig. S6). We concluded that *Tb*FlabarinL is not an essential gene for the viability of BSF *T. brucei* under cell culture conditions and *in vivo*.

### *Tb*FlabarinL is dispensable for developmental differentiation

**Figure 10:**
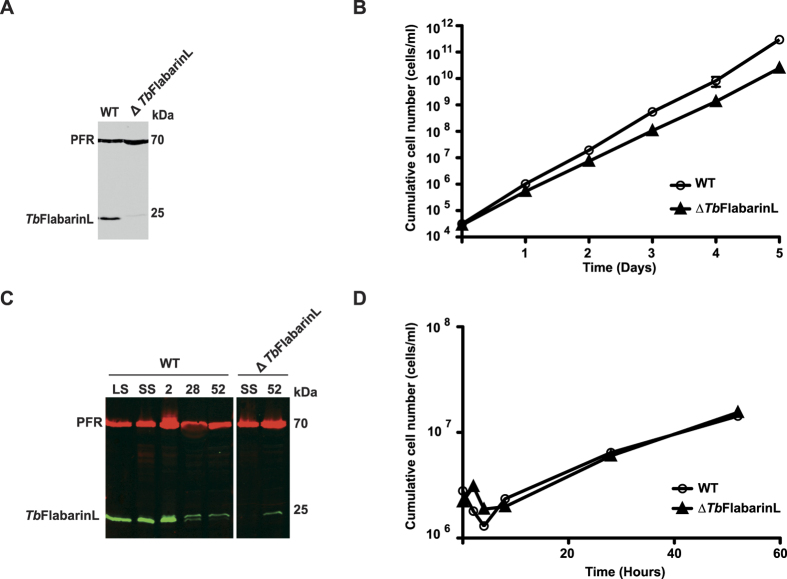
*Tb*FlabarinL deletion in pleomorphic BSF *T. brucei*. **(A)** Immunoblot analysis with *Tb*FlabarinL specific antibody verified the deletion of *Tb*FlabarinL. An anti-PFR immunoblot was used as a loading control. **(B)** Deletion of *Tb*FlabarinL causes a mild growth defect (N = 3). **(C)** Immunoblot analysis with *Tb*FlabarinL specific antibody confirmed the downregulation of *Tb*FlabarinL at different time points during differentiation from long slender (LS) to short stumpy (SS) and then 2, 28 and 52 hours after induction of differentiation. A weak additional band with the size of 25 kDa is detectable after induction of differentiation. **(D)** Pleomorphic trypanosomes were successfully differentiated from LS to PCF. There was no difference in the growth rate between the parental (WT) and ∆*Tb*FlabarinL cell line during and after differentiation (N = 1).

*Tb*FlabarinL has a very interesting expression profile during developmental differentiation. It is highly upregulated in LS and SS forms, still detectable during early differentiation but is downregulated below detection levels 48 h after induction of differentiation^19^. To test if *Tb*FlabarinL could be involved in BSF to PCF transition, a stable ∆*Tb*FlabarinL cell line was generated in a pleomorphic *T. brucei* strain. So-called monomorphic parasites, which we used in the previous experiments, are culture adapted and very convenient for reverse genetics, but initiation of differentiation is inherently inefficient and asynchronous. In pleomorphic field strains of *T. brucei*, differentiation is very efficient and both steps of the differentiation process (LS to SS and SS to PCF) can be monitored in cell culture. Furthermore, pleomorphic strains still respond to stumpy induction factor (SIF) with growth arrest *in vivo* and are therefore the better system to study differentiation cell biology and virulence *in vivo*. Hence, we generated pleomorphic ∆*Tb*FlabarinL trypanosomes in strain AnTat1.1 as described above. The loss of both *Tb*FlabarinL alleles was verified by integration PCR (Supplementary Fig. S4) and WB analysis (Fig. 10A). First, the growth of the ∆*Tb*FlabarinL cell line was compared to the parental cell line and only a mild growth phenotype was observed (Fig. 10B), which is in agreement with the result seen in the monomorphic ∆*Tb*FlabarinL cell line (Fig. 10). Second, the pleomorphic cells were grown to a high density to induce SS formation and then further differentiated for 52 hrs. WB analysis (Fig. 10C) confirmed the downregulation of *Tb*FlabarinL during differentiation seen in proteomic analysis^19^. ∆*Tb*FlabarinL and parental cells grew equally well during developmental differentiation (Fig. 10D), which suggests that *Tb*FlabarinL is dispensable for this process. A second very faint band migrating slightly higher than *Tb*FlabarinL was detected by anti-*Tb*FlabarinL antibody in the ∆*Tb*FlabarinL cell lines after induction of differentiation (Fig. 10C). The band is detectable in the WT pleomorphic cells during differentiation from SS to PCF at the time point 28 h post differentiation, when the *Tb*FlabarinL band becomes weaker. The same band is detectable during the differentiation of ∆*Tb*FlabarinL at the time point 52 h. To exclude that the anti-*Tb*FlabarinL antibody can recognize *Tb*Flabarin, WB analysis of a BSF cell line ectopically expressing Ty1 epitope-tagged *Tb*Flabarin was performed (Supplementary Fig. S5). Both anti-*Tb*FlabarinL and anti-Ty1 antibody could only detect one band, which supports the specificity of the *Tb*FlabarinL-specific antibody.

In summary, *Tb*FlabarinL is a result of a gene duplication of TbFlabarin, which occurred in African trypanosomes. We generated a *Tb*FlabarinL antibody and showed that it localizes to the flagellum. The results of IP experiments and a biochemical cell fractionation suggest a dual interaction of *Tb*FlabarinL with the flagellar membrane as well as with structural components of the flagellum such as the PFR. *Tb*FlabarinL is not essential for the growth and differentiation of *T. brucei* under cell culture conditions.

## Discussion

Trypanosomes must adapt to different host environments during their complex life cycle. Large-scale proteomic approaches provide information on changes at the cellular level in a systematic way. However, a detailed work on single components is necessary to understand the adaptation mechanisms on a molecular level. Here, we have performed a detailed characterization of a BSF stage-specific putative host adaptation factor Tb927.11.2400 identified in a SILAC-based comparative proteome study^16^. Tb927.11.2400 shares a 38% amino acid identity with *Tb*Flabarin (Tb927.11.2410), a PCF stage-specific^16^ flagellar BAR domain protein. The BAR domain is missed by the prediction software in Tb927.11.2400 and this is why we named it *Tb*Flabarin Like (*Tb*FlabarinL).

Flabarin was first described in *L. donovani* as a flagellar BAR domain protein^38^. It forms a helical structure from the base to the tip of the flagellum^38^. *In vitro* experiments suggested a morphogenetic and structural function since recombinant *Ld*Flabarin associates with liposomes and triggers tubule formation^38^. Recently, membranous nanotube formation was reported in *T. brucei*^70^. Those nanotubes appear to originate from the flagellar membrane and seem to dissociate into free extracellular vesicles (EVs). The protein composition of isolated EVs was determined by mass spectrometry and revealed the presence of *Tb*FlabarinL together with 155 other proteins^70^. *Tb*FlabarinL could be involved in nanotube/EVs formation or play a role in the fusion ability of the vesicles with other trypanosomes or erythrocytes or have an influence on the contents of the vesicles.

*Tb*FlabarinL and *Tb*Flabarin are both found adjacent on chromosome 11. We demonstrated that *Tb*FlabarinL is a result of a gene duplication event that occurred in African trypanosomes. However, in *T. congolense* and *T. equiperdum* a secondary loss of FlabarinL seems to have happened. This could be explained by the unique life cycles of these two species. In contrast to *T. brucei*, *T. congolense* is a strictly intravascular parasite and does not traverse different tissues^71^. *T. equiperdum* is essentially a tissue parasite in the reproductive system of *Equidae* family animals with a very low parasitemia in the blood and is transmitted venereally^72^. A second reason for the absence of FlabarinL in T*. congolense* and *T. equiperdum* could be the limited sequencing data available for both species. While the BSF stage upregulation suggested an essential role of *Tb*FlabarinL in the mammalian host, gene depletion and deletion experiments revealed that *Tb*FlabarinL is not an essential gene for the growth and differentiation of trypanosomes in cell culture. Infections in mice indicated that *Tb*FlabarinL is also dispensable *in vivo*. Based on the distribution of *Tb*FlabarinL across the different trypanosomatida species, we speculate that *Tb*FlabarinL was acquired as an adaptation to the lifestyle of different African trypanosomes, in particular to their ability to leave the bloodstream in the mammalian host and migrate through different tissues in the advanced stages of the infection. Alternatively, *Tb*FlabarinL might be important for developmental differentiation in the Tsetse fly. Unfortunately, we do not have information about changes in expression levels of *Tb*FlabarinL in different stages in the fly.

We generated a *Tb*FlabarinL-specific antibody and could detect *Tb*FlabarinL in the flagellum in a punctate pattern. Masking the N-terminal end of *Tb*FlabarinL with an epitope tag resulted in mislocalization of the protein. This provided evidence for the importance of the N-terminal sequence in targeting *Tb*FlabarinL to the flagellum. A closer inspection of the *Tb*FlabarinL N-terminus allowed us to hypothesize that the presence of positively charged lysines and potentially myristoylated glycine and palmitoylated cysteine might be important for targeting TbFlabarinL to the flagellar membrane as shown previously for FCaBP in *T. cruzi*^66^. A recent study of the flagellar arginine kinase 3 (AK3) summarizes so far known proteins such as *Tb*Caf17, *Tb*Caf24, *Tb*Caf44 which all share similar flagellar address with the FCaBP^36^. In addition, the BAR domain of *Ld*Flabarin mediates its association with the flagellar membrane^38^. A structural model of both *Tb*FlabarinL and *Tb*Flabarin predicted a coiled-coil helical architecture identical with *Ld*Flabarin despite a missing BAR domain *per se* and suggested a membrane association.

The punctate *Tb*FlabarinL localization pattern could be explained by a potential association of the *Tb*FlabarinL with discrete complexes in the flagellar membrane due to the presence of dual acylation. Dually acylated proteins have been shown to prefer association with membrane microdomains enriched in sphingolipids and cholesterol (reviewed in ref. 73). IP using anti-*Tb*FlabarinL specific antibody identified PAR1 as a possible interacting partner of *Tb*FlabarinL. PAR1 is a component of the PFR and unlike TbFlabarinL, it was detected in a comparative proteomic study^74^ together with 20 novel components of the PFR. Cell fractionation revealed that *Tb*FlabarinL is present not only in the detergent-soluble fraction but also it is tightly associated with the isolated flagella and cytoskeletal fractions containing the PFR, which is in concordance with the predicted flagellar membrane localization as well as the result of the IP experiment. We suggest that the association of *Tb*FlabarinL with the flagellar membrane might be only transient and function via a myristoyl switch mechanism, where the exposure of the myristate moiety could be ligand mediated as it is in the case of ADP ribosylation factor 1 (Arf1)^75^. The binding of Arf1 to the membrane is regulated by guanine nucleotide (GDP). When GDP is bound, the myristoylated N-terminal helix is sheltered in a hydrophobic groove of Arf1 and dissociates from the membrane^76^. Likewise when a ligand binds to TbFlabarinL, it might dissociate from the membrane and interact with the cytoskeletal components of the flagellum. While this manuscript was in preparation, Tetaud and colleagues published the characterization of *Tb*Flabarin in PCF^77^. They showed that TbFlabarin mRNA is mainly expressed in PCF and that the flagellar localization of the protein is dependent on two cysteines at the N-terminus, which might mediate the strong association of the protein with the parasite’s membrane in their palmitoylated form.

In this study we present a characterization of two flabarin orthologs in *T. brucei* with a major focus on *Tb*FlabarinL, which is BSF stage specific in contrast to PCF specific *Tb*Flabarin. Further research is still needed to fully understand the function of flabarins in trypanosomes.

## Additional Information

**How to cite this article**: Cicova, Z. *et al*. Two flagellar BAR domain proteins in *Trypanosoma brucei* with stage-specific regulation. *Sci. Rep.* **6**, 35826; doi: 10.1038/srep35826 (2016).

## Supplementary Material

Supplementary Information
