# Supplementary material for: Two flagellar BAR domain proteins in Trypanosoma brucei with stage-specific regulation
Source: Sci Rep. 2016 Oct 25;6:35826. doi: 10.1038/srep35826 (PMC5078803; doi:10.1038/srep35826)
Supplement: Supplementary Information [file srep35826-s1.pdf]

Two flagellar BAR domain proteins in *Trypanosoma brucei* with stage-specific regulation

Zdenka Cicova[1], Mario Dejung[2], Tomas Skalicky[3], Nicole Eisenhuth[1], Steffen Hanselmann[1], Brooke Morriswood[1], Luisa M. Figueiredo[4], Falk Butter[2], Christian J. Janzen[1]

[1] Department of Cell & Developmental Biology, Biocenter, University of Würzburg, Würzburg, Germany

[2] Institute of Molecular Biology (IMB), Mainz, Germany

[3] Laboratory of Molecular Biology of Protists, Institute of Parasitology Biology Centre, Czech Academy of Science, and Faculty of Sciences, University of South Bohemia Ceske Budejovice, Czech Republic

[4] Instituto de Medicina Molecular, Faculdade de Medicina, Universidade de Lisboa, Lisboa 1649-028, Portugal

Organism	Ortholog	Accession number
T. brucei brucei	*Tb*Flabarin	Tb927.11.2410
T. brucei gambiense	*Tb*Flabarin	Tbg927.11.2210
T. congolense	*Tb*Flabarin	TcIL3000.11.2210
T. cruzi Dm28c	*Tb*Flabarin	ESS66808.1
T. cruzi CL Brener	*Tb*Flabarin	TcCLB.506125.20
T. equiperdum	*Tb*Flabarin	CZPT01000935.1
T. grayi	*Tb*Flabarin	XP_009315916.1
T. vivax	*Tb*Flabarin	TvY486_0013090
L. panamensis	*Tb*Flabarin	XP_010700258.1
L. braziliensis	*Tb*Flabarin	XP_001565941.1
L. mexicana	*Tb*Flabarin	XP_003876746.1
L. infantum	*Tb*Flabarin	XP_001466397.1
L. major Friedlin	*Tb*Flabarin	XP_003721979.1
L. seymouri	*Tb*Flabarin	KPI89862.1
L. pyrrhocoris	*Tb*Flabarin	H10_07_2100
A. deanei	*Tb*Flabari	EPY38881.1
C. fasciculata	*Tb*Flabarin	CFAC1_230031800
L. donovani	*Tb*Flabarin	LdBPK_271630.1
L. tarentolae	*Tb*Flabarin	P27.1790
L. tropica	*Tb*Flabarin	LTRL590_270024000
T. evansi	*Tb*Flabarin	TevSTIB805.11.01.2480
Phytomonas sp. EM1	*Tb*Flabarin	CCW60919.1
Phytomonas sp. Hart1	*Tb*Flabarin	CCW71432.1
E. monterogeii	*Tb*Flabarin	EMOLV88_270021800
P. confusum	*Tb*Flabarin	KU375192
C. acanthocephali	*Tb*Flabarin	AUXI01000516.1
H. muscarum	*Tb*Flabarin	AUXJ01000865.1
S. galati	*Tb*Flabarin	AUXN01000492.1
S. oncopelti	*Tb*Flabarin	AUXK01006077.1
A. desouzai	*Tb*Flabarin	AUXL01001281.1
S. culicis	*Tb*Flabarin	AUXH01000047.1
T. brucei brucei	*Tb*FlabarinL	Tb927.11.2400
T. brucei gambiense	*Tb*FlabarinL	Tbg972.11.2650
T. vivax	*Tb*FlabarinL	TvY486_1102400
T. evansi	*Tb*FlabarinL	TevSTIB805.11.01.2450

Supplementary table 1. List of proteins used for the phylogenetic analysis

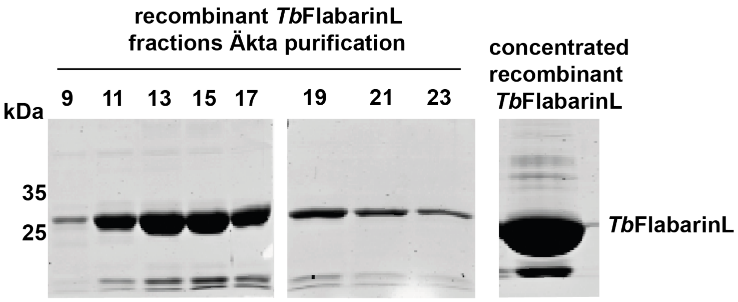

Supplementary figure S1. Expression of recombinant *Tb*FlabarinL.
Recombinant *Tb*FlabarinL fused to a 10x His-tag with a Gly-Ala-Gly linker at the N-terminus was expressed and purified from *E. coli* using an Äkta FPLC and a HisTrap FF crude column. The indicated fractions containing the purified protein were separated by SDS PAGE and stained with Coomassie brilliant blue. Fractions containing recombinant *Tb*FlabarinL were pooled, dialyzed in phosphate buffered saline and concentrated using a centrifugal Amicon filter unit with a 10 kDa cut off (concentrated recombinant *Tb*FlabarinL).

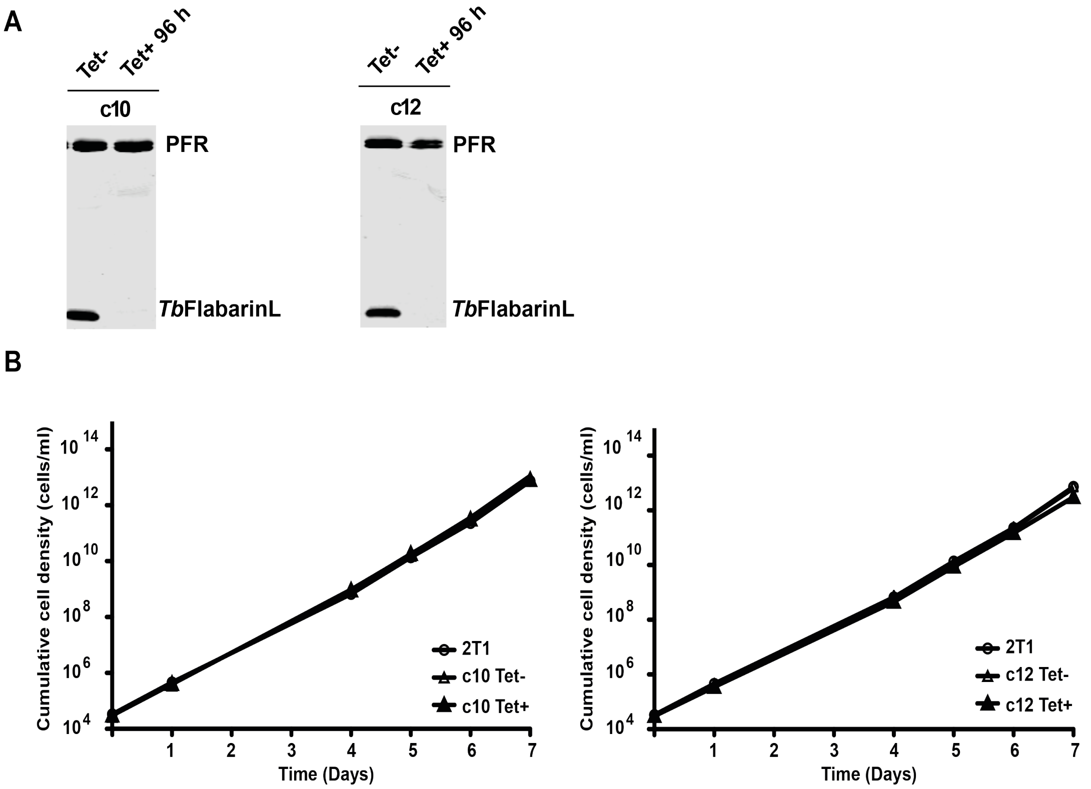

Supplementary figure S2. RNAi-mediated depletion of *Tb*FlabarinL in BSF *T. brucei* clones c10 and c12. **(A)** *Tb*FlabarinL protein was reduced below the detection level 96 h after induction of RNAi as determined by *Tb*FlabarinL specific antibody immunoblot. An immunoblot with anti-PFR antibodies was used as a loading control. **(B)** Depletion of *Tb*FlabarinL by RNAi had no effect on the growth of BSF *T. brucei* in vitro. Cumulative growth curves (N=1) of two different clones c10 and c12 are shown.

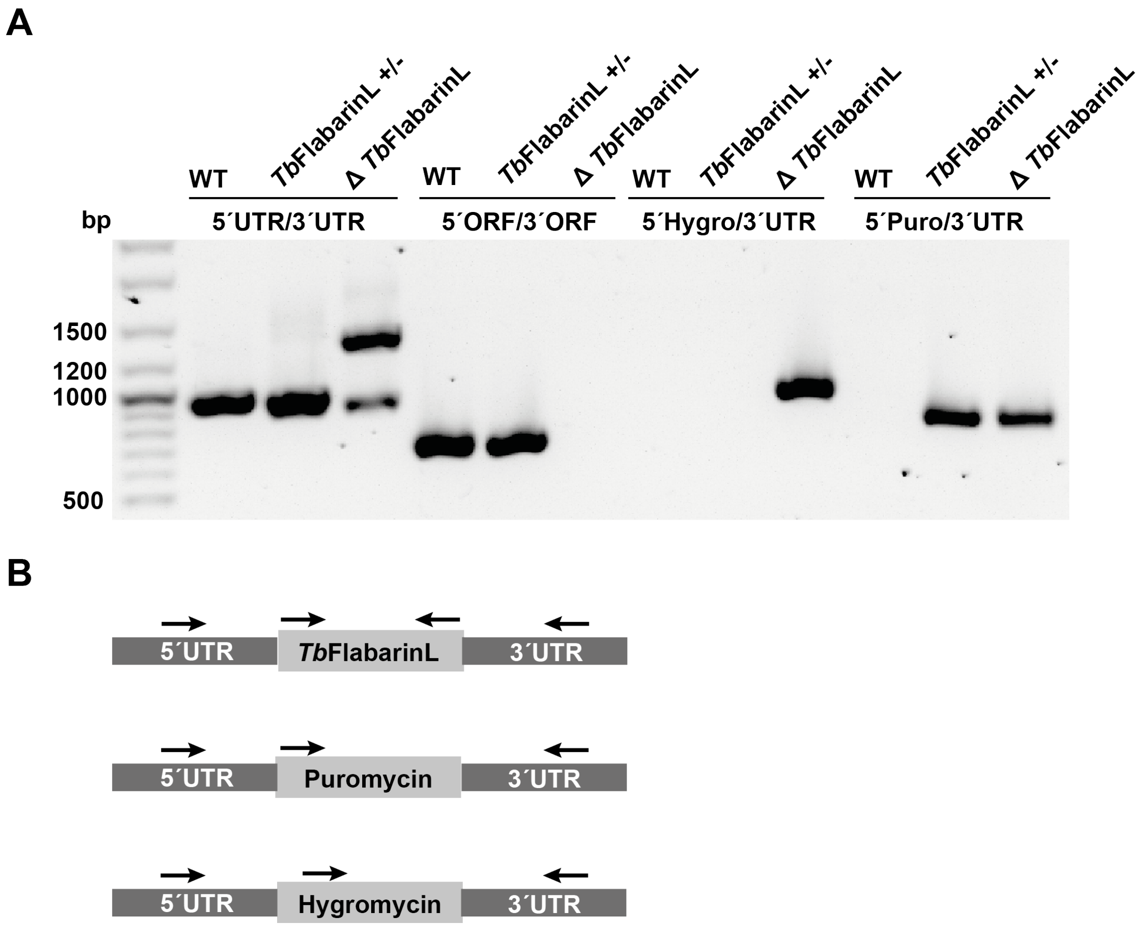

Supplementary Fig S3. Deletion of *Tb*FlabarinL in monomorphic BSF *T. brucei.* (A) Integration PCR verified the correct integration of puromycin N-acetyl-transferase and hygromycin phosphotransferase ORFs that replaced both alleles of the *Tb*FlabarinL gene. WT, *Tb*FlabarinL +/- and Δ*Tb*FlabarinL cell lines were tested using primers binding in the 5´and 3´UTR, 5´and 3´ORF and within the puromycin and hygromycin resistance ORFs. **(B)** A scheme depicting the primers annealing in the 5´and 3´UTR, 5´and 3´ORF and within the puromycin and hygromycin resistance cassettes.

A

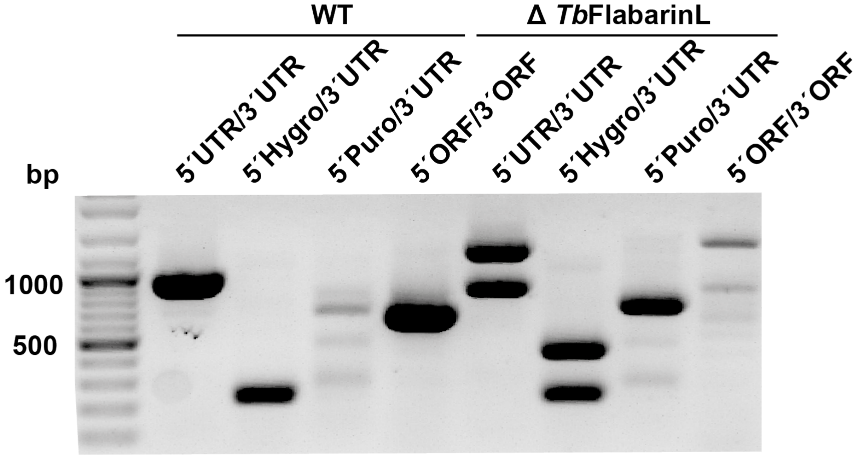

B

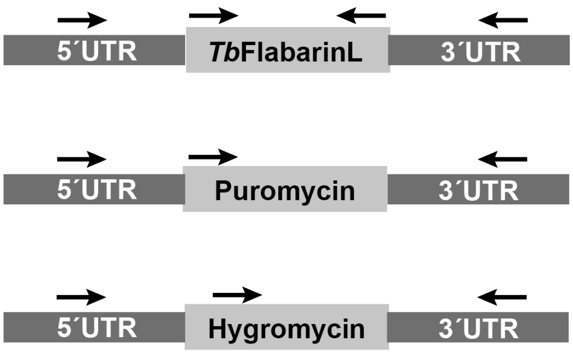

Supplementary Fig S4. Deletion of *Tb*FlabarinL in pleomorphic BSF *T. brucei.* (A) Integration PCR validated the correct integration of puromycin N-acetyl-transferase and hygromycin phosphotransferase ORFs that replaced both alleles of *Tb*FlabarinL gene. WT and Δ*Tb*FlabarinL cell lines were tested using primers binding in the 5´and 3´UTR, 5´and 3´ORF and within the puromycin and hygromycin resistance ORFs. **(B)** Primers annealing in the 5´and 3´UTR, 5´and 3´ORF and within the puromycin and hygromycin ORFs were used.

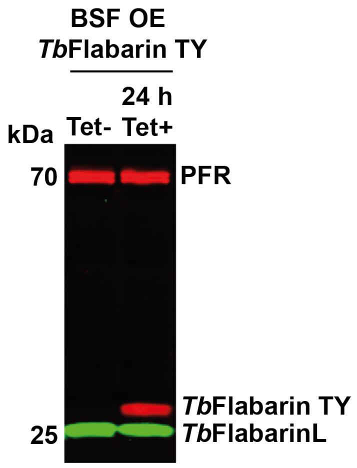

Supplementary figure S5. Anti-*Tb*FlabarinL antibody recognizes *Tb*FlabarinL but not *Tb*Flabarin.
Immunoblot analysis using *Tb*FlabarinL specific antibody (green), anti-Ty1 (red) and anti-PFR (red) antibodies showed that *Tb*FlabarinL specific antibody recognized only *Tb*FlabarinL. The *Tb*FlabarinL-specific antibody does not cross-react with the *Tb*Flabarin even though the two proteins share 38% identity at the amino acid level.

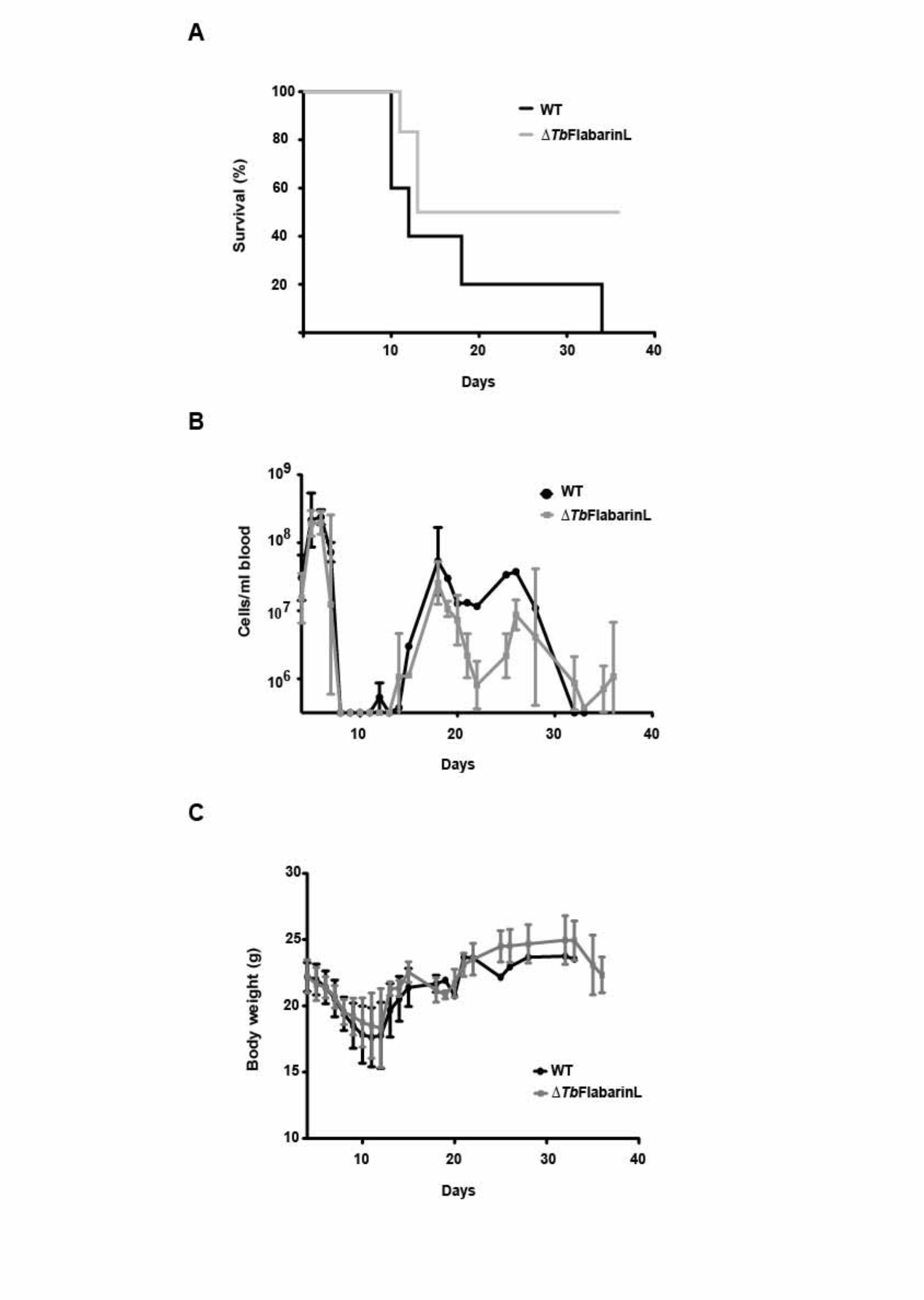

Supplementary figure S6. **Infection of mice**.
(A) Survival curve **(B)** parasitemia and **(C)** body weight of C57BL/6 mice infected with Δ*Tb*FlabarinL pleomorphic *T. brucei* AnTat1.1E SmOx compared to mice infected with the parental *T. brucei* AnTat1.1E SmOx parasites (WT). Six mice were infected with the Δ*Tb*FlabarinL and 5 mice were infected with the parental cells (WT) at day 0 (intraperitoneal injection of 2000 parasites per mouse).
